# rs2735383, located at a microRNA binding site in the 3’UTR of *NBS1*, is not associated with breast cancer risk

**DOI:** 10.1038/srep36874

**Published:** 2016-11-15

**Authors:** Jingjing Liu, Ivona Lončar, J. Margriet Collée, Manjeet K. Bolla, Joe Dennis, Kyriaki Michailidou, Qin Wang, Irene L. Andrulis, Monica Barile, Matthias W. Beckmann, Sabine Behrens, Javier Benitez, Carl Blomqvist, Bram Boeckx, Natalia V. Bogdanova, Stig E. Bojesen, Hiltrud Brauch, Paul Brennan, Hermann Brenner, Annegien Broeks, Barbara Burwinkel, Jenny Chang-Claude, Shou-Tung Chen, Georgia Chenevix-Trench, Ching Y. Cheng, Ji-Yeob Choi, Fergus J. Couch, Angela Cox, Simon S. Cross, Katarina Cuk, Kamila Czene, Thilo Dörk, Isabel dos-Santos-Silva, Peter A. Fasching, Jonine Figueroa, Henrik Flyger, Montserrat García-Closas, Graham G. Giles, Gord Glendon, Mark S. Goldberg, Anna González-Neira, Pascal Guénel, Christopher A. Haiman, Ute Hamann, Steven N. Hart, Mikael Hartman, Sigrid Hatse, John L. Hopper, Hidemi Ito, Anna Jakubowska, Maria Kabisch, Daehee Kang, Veli-Matti Kosma, Vessela N. Kristensen, Loic Le Marchand, Eunjung Lee, Jingmei Li, Artitaya Lophatananon, Jan Lubinski, Arto Mannermaa, Keitaro Matsuo, Roger L. Milne, NBCS Collaborators, Kristine K. Sahlberg, Lars Ottestad, Rolf Kåresen, Anita Langerød, Ellen Schlichting, Marit Muri Holmen, Toril Sauer, Vilde Haakensen, Olav Engebråten, Bjørn Naume, Cecile E. Kiserud, Kristin V. Reinertsen, åslaug Helland, Margit Riis, Ida Bukholm, Per Eystein Lønning, Anne-Lise Børresen-Dale, Grethe I. Grenaker Alnæs, Susan L. Neuhausen, Heli Nevanlinna, Nick Orr, Jose I. A. Perez, Julian Peto, Thomas C. Putti, Katri Pylkäs, Paolo Radice, Suleeporn Sangrajrang, Elinor J. Sawyer, Marjanka K. Schmidt, Andreas Schneeweiss, Chen-Yang Shen, Martha J. Shrubsole, Xiao-Ou Shu, Jacques Simard, Melissa C. Southey, Anthony Swerdlow, Soo H. Teo, Daniel C. Tessier, Somchai Thanasitthichai, Ian Tomlinson, Diana Torres, Thérèse Truong, Chiu-Chen Tseng, Celine Vachon, Robert Winqvist, Anna H. Wu, Drakoulis Yannoukakos, Wei Zheng, Per Hall, Alison M. Dunning, Douglas F. Easton, Maartje J. Hooning, Ans M. W. van den Ouweland, John W. M. Martens, Antoinette Hollestelle

**Affiliations:** 1 Department of Medical Oncology, Family Cancer Clinic, Erasmus MC Cancer Institute, Rotterdam, The Netherlands; 2 Department of Clinical Genetics, Erasmus University Medical Center, Rotterdam, The Netherlands; 3 Centre for Cancer Genetic Epidemiology, Department of Public Health and Primary Care, University of Cambridge, Cambridge, UK; 4 Department of Electron Microscopy/Molecular Pathology, The Cyprus Institute of Neurology and Genetics, Nicosia, Cyprus; 5 Fred A. Litwin Center for Cancer Genetics, Lunenfeld-Tanenbaum Research Institute of Mount Sinai Hospital, Toronto, Canada; 6 Department of Molecular Genetics, University of Toronto, Toronto, Canada; 7 Division of Cancer Prevention and Genetics, Istituto Europeo di Oncologia, Milan, Italy; 8 Department of Gynaecology and Obstetrics, University Hospital Erlangen, Friedrich-Alexander University Erlangen-Nuremberg, Comprehensive Cancer Center Erlangen-EMN, Erlangen, Germany; 9 Division of Cancer Epidemiology, German Cancer Research Center (DKFZ), Heidelberg, Germany; 10 University Cancer Center Hamburg (UCCH), University Medical Center Hamburg-Eppendorf, Hamburg, Germany; 11 Human Cancer Genetics Program, Spanish National Cancer Research Centre, Madrid, Spain; 12 Centro de Investigación en Red de Enfermedades Raras (CIBERER), Valencia, Spain; 13 Department of Oncology, Helsinki University Hospital, University of Helsinki, Helsinki, Finland; 14 Vesalius Research Center, VIB, Leuven, Belgium; 15 Laboratory for Translational Genetics, Department of Oncology, University of Leuven, Leuven, Belgium; 16 Department of Radiation Oncology, Hannover Medical School, Hannover, Germany; 17 Gynaecology Research Unit, Hannover Medical School, Hannover, Germany; 18 N.N. Alexandrov Research Institute of Oncology and Medical Radiology, Minsk, Belarus; 19 Copenhagen General Population Study, Herlev and Gentofte Hospital, Copenhagen University Hospital, Herlev, Denmark; 20 Department of Clinical Biochemistry, Herlev and Gentofte Hospital, Copenhagen University Hospital, Herlev, Denmark; 21 Faculty of Health and Medical Sciences, University of Copenhagen, Copenhagen, Denmark; 22 Dr. Margarete Fischer-Bosch-Institute of Clinical Pharmacology, Stuttgart, Germany; 23 University of Tübingen, Tübingen, Germany; 24 German Cancer Consortium (DKTK), German Cancer Research Center (DKFZ), Heidelberg, Germany; 25 International Agency for Research on Cancer, Lyon, France; 26 Division of Clinical Epidemiology and Aging Research, German Cancer Research Center (DKFZ), Heidelberg, Germany; 27 Division of Preventive Oncology, German Cancer Research Center (DKFZ) and National Center for Tumor Diseases (NCT), Heidelberg, Germany; 28 Division of Molecular Pathology, The Netherlands Cancer Institute - Antoni van Leeuwenhoek Hospital, Amsterdam, The Netherlands; 29 Department of Obstetrics and Gynecology, University of Heidelberg, Heidelberg, Germany; 30 Molecular Epidemiology Group, C080, German Cancer Research Center (DKFZ), Heidelberg, Germany; 31 Department of Surgery, Changhua Christian Hospital, Changhua, Taiwan; 32 Department of Genetics and Computational Biology, QIMR Berghofer Medical Research Institute, Brisbane, Australia; 33 Singapore Eye Research Institute and Singapore National Eye Center, Singapore, Singapore; 34 Department of Ophthalmology, Yong Loo Lin School of Medicine, National University of Singapore and National University Health System, Singapore, Singapore; 35 Duke-NUS Graduate Medical School, Singapore, Singapore; 36 Department of Biomedical Sciences, Seoul National University College of Medicine, Seoul, Korea; 37 Cancer Research Institute, Seoul National University, Seoul, Korea; 38 Department of Laboratory Medicine and Pathology, Mayo Clinic, Rochester, MN, USA; 39 Academic Unit of Molecular Oncology, Department of Oncology and Metabolism, University of Sheffield, Sheffield, UK; 40 Academic Unit of Pathology, Department of Neuroscience, University of Sheffield, Sheffield, UK; 41 Department of Medical Epidemiology and Biostatistics, Karolinska Institutet, Stockholm, Sweden; 42 Department of Non-Communicable Disease Epidemiology, London School of Hygiene and Tropical Medicine, London, UK; 43 David Geffen School of Medicine, Department of Medicine Division of Hematology and Oncology, University of California at Los Angeles, Los Angeles, CA, USA; 44 Usher Institute of Population Health Sciences and Informatics, The University of Edinburgh Medical School, Edinburgh, UK; 45 Division of Cancer Epidemiology and Genetics, National Cancer Institute, Rockville, MD, USA; 46 Department of Breast Surgery, Herlev and Gentofte Hospital, Copenhagen University Hospital, Herlev, Denmark; 47 Cancer Epidemiology Centre, Cancer Council Victoria, Melbourne, Australia; 48 Centre for Epidemiology and Biostatistics, Melbourne School of Population and Global health, The University of Melbourne, Melbourne, Australia; 49 Department of Medicine, McGill University, Montreal, Canada; 50 Division of Clinical Epidemiology, Royal Victoria Hospital, McGill University, Montreal, Canada; 51 Cancer & Environment Group, Center for Research in Epidemiology and Population Health (CESP), INSERM, University Paris-Sud, University Paris-Saclay, Villejuif, France; 52 Department of Preventive Medicine, Keck School of Medicine, University of Southern California, Los Angeles, CA, USA; 53 Molecular Genetics of Breast Cancer, German Cancer Research Center (DKFZ), Heidelberg, Germany; 54 Department of Health Sciences Research, Mayo Clinic, Rochester, MN, USA; 55 Saw Swee Hock School of Public Health, National University of Singapore, Singapore, Singapore; 56 Department of Surgery, National University Health System, Singapore, Singapore; 57 Leuven Multidisciplinary Breast Center, Department of Oncology, Leuven Cancer Institute, University Hospitals Leuven, Leuven, Belgium; 58 Division of Epidemiology and Prevention, Aichi Cancer Center Research Institute, Nagoya, Japan; 59 Department of Epidemiology, Nagoya University Graduate School of Medicine, Nagoya, Japan; 60 Department of Genetics and Pathology, Pomeranian Medical University, Szczecin, Poland; 61 Department of Preventive Medicine, Seoul National University College of Medicine, Seoul, Korea; 62 Translational Cancer Research Area, University of Eastern Finland, Kuopio, Finland; 63 Institute of Clinical Medicine, Pathology and Forensic Medicine, University of Eastern Finland, Kuopio, Finland; 64 Imaging Center, Department of Clinical Pathology, Kuopio University Hospital, Kuopio, Finland; 65 Department of Cancer Genetics, Institute for Cancer Research, Oslo University Hospital Radiumhospitalet, Oslo, Norway; 66 Department of Clinical Molecular Biology, Oslo University Hospital, University of Oslo, Oslo, Norway; 67 Epidemiology Program, University of Hawaii Cancer Center, Honolulu, HI, USA; 68 Division of Health Sciences, Warwick Medical School, Warwick University, Coventry, UK; 69 Institute of Population Health, University of Manchester, Manchester, UK; 70 Division of Molecular Medicine, Aichi Cancer Center Research Institute, Nagoya, Japan; 71 Department of Population Sciences, Beckman Research Institute of City of Hope, Duarte, CA, USA; 72 Department of Obstetrics and Gynecology, Helsinki University Hospital, University of Helsinki, Helsinki, Finland; 73 Division of Breast Cancer Research, The Institute of Cancer Research, London, UK; 74 Servicio de Cirugía General y Especialidades, Hospital Monte Naranco, Oviedo, Spain; 75 Department of Pathology, Yong Loo Lin School of Medicine, National University of Singapore, Singapore, Singapore; 76 Laboratory of Cancer Genetics and Tumor Biology, Cancer and Translational Medicine Research Unit, Biocenter Oulu, University of Oulu, Oulu, Finland; 77 Laboratory of Cancer Genetics and Tumor Biology, Northern Finland Laboratory Centre NordLab, Oulu, Finland; 78 Unit of Molecular Bases of Genetic Risk and Genetic Testing, Department of Preventive and Predictive Medicine, Fondazione IRCCS (Istituto Di Ricovero e Cura a Carattere Scientifico) Istituto Nazionale dei Tumori (INT), Milan, Italy; 79 National Cancer Institute, Bangkok, Thailand; 80 Research Oncology, Guy’s Hospital, King’s College London, London, UK; 81 Division of Psychosocial Research and Epidemiology, The Netherlands Cancer Institute - Antoni van Leeuwenhoek hospital, Amsterdam, The Netherlands; 82 National Center for Tumor Diseases, University of Heidelberg, Heidelberg, Germany; 83 School of Public Health, China Medical University, Taichung, Taiwan; 84 Taiwan Biobank, Institute of Biomedical Sciences, Academia Sinica, Taipei, Taiwan; 85 Division of Epidemiology, Department of Medicine, Vanderbilt Epidemiology Center, Vanderbilt-Ingram Cancer Center, Vanderbilt University School of Medicine, Nashville, TN, USA; 86 Genomics Center, Centre Hospitalier Universitaire de Québec Research Center, Laval University, Québec City, Canada; 87 Genetic Epidemiology Laboratory, Department of Pathology, The University of Melbourne, Melbourne, Australia; 88 Division of Genetics and Epidemiology, The Institute of Cancer Research, London, UK; 89 Cancer Research Malaysia, Subang Jaya, Selangor, Malaysia; 90 Breast Cancer Research Unit, Cancer Research Institute, University Malaya Medical Centre, Kuala Lumpur, Malaysia; 91 McGill University and Génome Québec Innovation Centre, Montréal, Canada; 92 Research and Technology Assessment Department, National Cancer Institute, Thailand; 93 Wellcome Trust Centre for Human Genetics and Oxford NIHR Biomedical Research Centre, University of Oxford, Oxford, UK; 94 Institute of Human Genetics, Pontificia Universidad Javeriana, Bogota, Colombia; 95 Molecular Diagnostics Laboratory, INRASTES, National Centre for Scientific Research “Demokritos”, Athens, Greece; 96 Centre for Cancer Genetic Epidemiology, Department of Oncology, University of Cambridge, Cambridge, UK; 97 Cancer Genomics Netherlands, Utrecht, The Netherlands; 98 Department of Research, Vestre Viken, Drammen, Norway.; 99 Institute of Clinical Medicine, Faculty of Medicine, University of Oslo, Oslo, Norway.; 100 Department of Breast and Endocrine Surgery, Oslo University Hospital, Ullevål, Oslo, Norway.; 101 Department of Radiology and Nuclear Medicine, Oslo University Hospital Radiumhospitalet, Oslo, Norway.; 102 Department of Pathology, Akershus University Hospital, L⊘renskog, Norway.; 103 Department of Tumor Biology, Institute for Cancer Research, Oslo University Hospital Radiumhospitalet, Oslo, Norway.; 104 Department of Oncology, Division of Surgery and Cancer and Transplantation Medicine, Oslo University Hospital Radiumhospitalet, Oslo, Norway.; 105 National Advisory Unit on Late Effects after Cancer Treatment, Division of Surgery and Cancer and Transplantation Medicine, Oslo University Hospital, Oslo, Norway.; 106 Department of Oncology, Oslo University Hospital Ullevål, Oslo, Norway.; 107 Department of Breast-Endocrine Surgery, Akershus University Hospital, Oslo, Norway.; 108 Section of Oncology, Institute of Medicine, University of Bergen, Bergen, Norway.; 109 Department of Oncology, Haukeland University Hospital, Bergen, Norway.

## Abstract

NBS1, also known as NBN, plays an important role in maintaining genomic stability. Interestingly, rs2735383 G > C, located in a microRNA binding site in the 3′-untranslated region (UTR) of *NBS1*, was shown to be associated with increased susceptibility to lung and colorectal cancer. However, the relation between rs2735383 and susceptibility to breast cancer is not yet clear. Therefore, we genotyped rs2735383 in 1,170 familial non-*BRCA1/2* breast cancer cases and 1,077 controls using PCR-based restriction fragment length polymorphism (RFLP-PCR) analysis, but found no association between rs2735383CC and breast cancer risk (OR = 1.214, 95% CI = 0.936–1.574, *P* = 0.144). Because we could not exclude a small effect size due to a limited sample size, we further analyzed imputed rs2735383 genotypes (*r*^*2*^ > 0.999) of 47,640 breast cancer cases and 46,656 controls from the Breast Cancer Association Consortium (BCAC). However, rs2735383CC was not associated with overall breast cancer risk in European (OR = 1.014, 95% CI = 0.969–1.060, *P* = 0.556) nor in Asian women (OR = 0.998, 95% CI = 0.905–1.100, *P* = 0.961). Subgroup analyses by age, age at menarche, age at menopause, menopausal status, number of pregnancies, breast feeding, family history and receptor status also did not reveal a significant association. This study therefore does not support the involvement of the genotype at *NBS1* rs2735383 in breast cancer susceptibility.

The DNA damage response (DDR) pathway maintains the stability of the human genome via a complex network of pathways integrating signal transduction, regulation of the cell cycle and repair of DNA. Double-strand breaks (DSBs), a particularly severe form of DNA damage, arise as a consequence of cell replication, programmed DNA rearrangements (*i.e.* meiosis and VDJ recombination) and exposure to carcinogens. When left unrepaired, DSBs may cause genomic instability, cell death and cancer^1^,^2^. In fact, mutations in genes involved in DSB repair, but also in the DDR pathway in general, are involved in the etiology of many human cancers. The two major repair pathways that mediate the repair of DSBs are the template-mediated homologous recombination repair pathway and the more error-prone non-homologous end-joining pathway^3^,^4^. The MRE11/RAD50/NBS1 complex is an important regulator of DSB repair through these pathways as this complex not only acts as a sensor of DSBs, but also recruits and activates the ATM protein to the break and activates it^5^. Activation of ATM, the central mediator of response to DSBs, initiates a cascade of signaling pathways involved in cell cycle checkpoint control, DNA repair and, when necessary, apoptosis by phosphorylation of p53, CHEK2, BRCA1, FANCD2 and NBS1 amongst others^6^.

The DDR plays an important role in susceptibility to breast cancer. In fact, all of the currently identified high- and moderate-risk breast cancer genes (*i.e. BRCA1, BRCA2, CHEK2, ATM, NBS1* and *PALB2)* are involved in DNA repair^7^,^8^. As the majority of familial breast cancer risk is not yet attributable to known risk genes, this makes other genes encoding proteins involved in the DDR pathway attractive candidates for breast cancer susceptibility genes. The recent identification of the early DNA damage response gene *MCPH1*as a novel breast cancer susceptibility gene illustrates that this hypothesis still holds^9^.

In this respect, the *NBS1* gene is located at chromosome 8q21 and bi-allelic germline mutations in *NBS1* cause the chromosomal instability syndrome Nijmegen breakage syndrome^10^. In addition, heterozygous carriers of *NBS1* mutations are at an increased risk to develop several types of cancer^11^. The *NBS1* c.657del5 founder mutation is the most prevalent mutation implicated in Nijmegen breakage syndrome (*i.e.* 90%) and has its origin in the Slavic population^12^. The mutation confers an overall 2.5- to 3-fold increased cancer risk and is associated with increased risk for breast cancer, prostate cancer and lymphoma specifically^13^. Two other *NBS1* mutations implicated in Nijmegen breakage syndrome are p.I171V and p.R215W. Although both mutations associate with an overall cancer risk of 4-fold and 2-fold, respectively, there does not seem to be an increased risk to develop breast cancer specifically^13^.

Besides the rare Nijmegen breakage syndrome-associated mutations, two common variants in *NBS1 (i.e.* p.E185Q; rs1805794 and c.2265 + 541G > C; rs2735383) have also been reported to be associated with risks for several cancer types. Recent meta-analyses for rs1805794 have, however, shown that this variant does not associate with breast cancer risk^13^,^14^,^15^,^16^, while associations with lung cancer and urinary system cancer are still inconclusive^13^,^16^,^17^,^18^. The functional variant rs2735383, localized in in the 3′ UTR of *NBS1*, has been shown to modulate the binding ability of microRNA-629 in lung cancer cells and microRNA-509-5p in colorectal cancer cells, affect *NBS1* transcriptional activity and decrease *NBS1* mRNA and NBS1 protein levels^19^,^20^. Although rs2735383 has been associated with an increased risk of lung cancer and colorectal cancer^13^,^20^, its association with breast cancer risk is yet unclear. For this reason, we assessed whether *NBS1* rs2735383 is associated with breast cancer risk in the Rotterdam Breast Cancer Study (RBCS) by RFLP-PCR and in 45 studies of BCAC through imputation of the iCOGS array^24^.

## Results

To evaluate the association between *NBS1* rs2735383 and breast cancer risk, we analyzed *NBS1* rs2735383 by RFLP-PCR in 1,269 non-*BRCA1/2* familial breast cancer patients and 1,159 controls from RBCS. Since genetic risk factors are usually enriched in familial/early-onset breast cancer cases, specifically selecting these breast cancer patients improves statistical power. Among the cases, 516 had the GG genotype, 507 had the GC genotype and 147 had the CC genotype at rs2735383 (minor allele frequency (MAF) = 0.342). Among the controls, 462 had the GG genotype, 501 had the GC genotype and 114 had the CC genotype (MAF = 0.338). For both cases and controls, the genotypes of rs2735383 were in Hardy-Weinberg equilibrium (HWE). Because rs2735383 CC was associated with an increased risk of lung cancer and colorectal cancer under a recessive genetic model^13^,^20^, we analyzed the association of rs2735383 with breast cancer in a similar way. However, rs2735383 was not significantly associated with the risk of breast cancer (OR = 1.214, 95% CI = 0.936–1.574, *P* = 0.144; Table 1). In this respect, the lung cancer risk conferred by the rs2735383 CC genotype had been associated with an OR of 1.28 (95% CI = 1.21–1.46, *P* < 0.001), whereas the colorectal cancer risk had been associated with an OR of 1.55 (95% CI = 1.27–1.94, *P* < 10^−4^)^13^,^20^. Here, we do not observe a similar effect size for breast cancer as for lung and colorectal cancer. However, RBCS is underpowered to detect effect sizes smaller than those observed for lung cancer *(i.e.* OR < 1.28). Therefore, we cannot exclude rs2735383 CC is associated with breast cancer, but confers a smaller risk.

For this reason we analyzed *NBS1* rs2735383 in BCAC studies through imputation. Since we had data available for RBCS on rs2735383 from both the PCR-based RFLP and from imputation, we first evaluated the concordance between the two methods. In total, from 1,313 samples (*i.e.* 646 cases and 667 controls) we had genotypes for rs2738353 available from both RFLP-PCR and imputation. Importantly, the agreement between the two methods was 97.1% (*i.e.* concordance in 1,275 of 1,313 samples, *r*^*2*^ = 0.933) and was similar among cases and controls (*i.e.* 98.1% versus 96.1%). Moreover, case-control ORs for imputed data were comparable to ORs obtained by RFLP-PCR (OR = 1.14, 95% CI = 0.80–1.62 versus OR = 1.17, 95% CI = 0.83–1.66). Therefore, we used the imputed data on rs2735383 to evaluate further its association with breast cancer risk.

**Figure 1:**
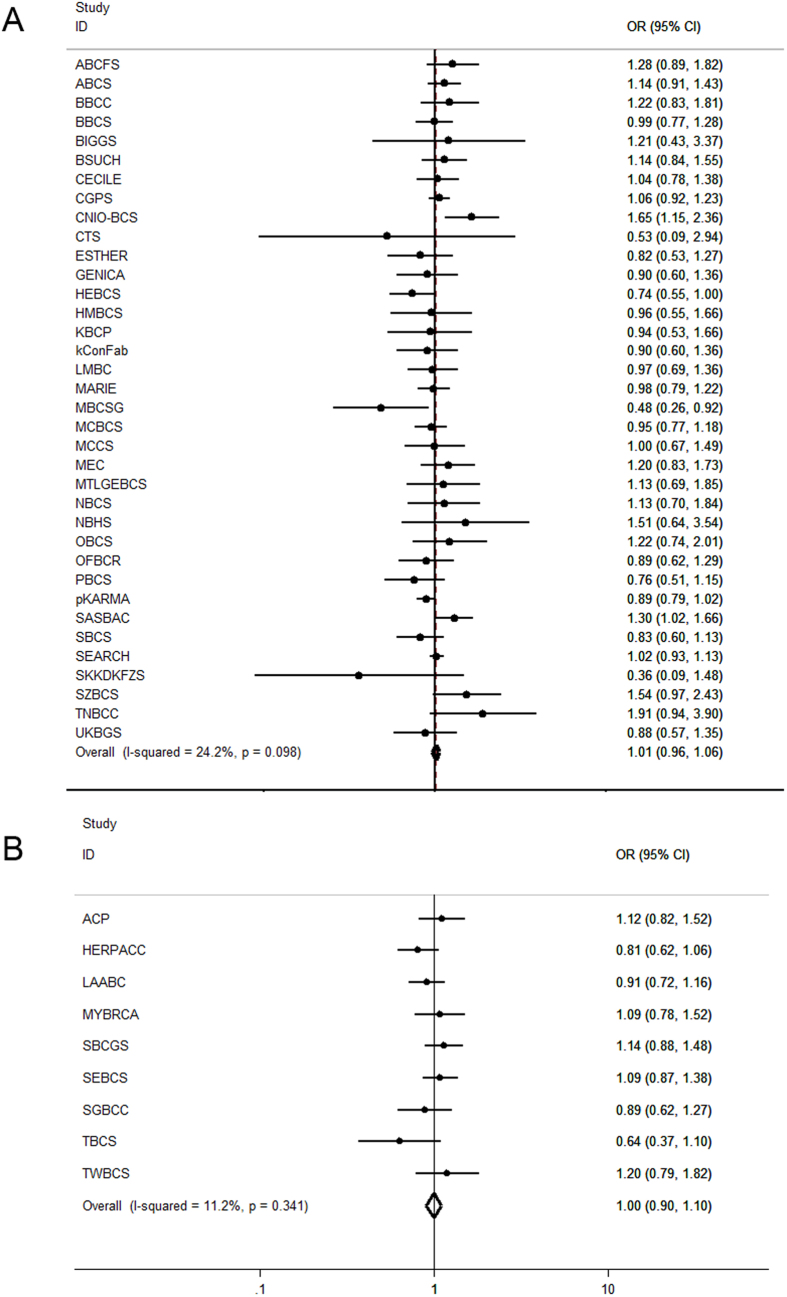
Forest plots for the association between rs2735383 and breast cancer risk. (**A**) for the 36 European BCAC studies and (**B**) for the nine Asian BCAC studies. Study-specific ORs (squares) were from a recessive genetic model and adjusted by age and PCs. Overall or pooled ORs (diamonds) were from a fixed-effects meta-analysis.

For the overall analysis in Europeans we had 41,915 cases and 40,042 controls available from 36 case-control studies. However, rs2735383 was not associated with breast cancer risk in Europeans, neither under a recessive genetic model (OR = 1.014, 95% CI = 0.969–1.060, *P* = 0.556; Table 2 and Fig. 1), nor under a dominant (OR = 1.006, 95% CI = 0.978–1.035, *P* = 0.684; Table 2) or additive model (per allele OR = 1.000, 95% CI = 0.979–1.021, *P* = 0.984; Table 2). Because the association with increased lung and colorectal cancer risk was observed in the Asian population^13^,^20^, we also performed the same analysis in the nine Asian BCAC studies. In total, we had 5,725 cases and 6,614 controls available for this analysis from nine case-control studies. Also in Asians we did not find any association between rs2735383 and breast cancer risk for either the recessive (OR = 0.998, 95% CI = 0.905–1.100, *P* = 0.961; Table 2 and Fig. 1), dominant (OR = 0.995, 95% CI = 0.922–1.074, *P* = 0.900; Table 2) or additive genetic model (per allele OR = 0.997, 95% CI = 0.946–1.050, *P* = 0.911; Table 2). These results imply that *NBS1* rs2735383 is not associated with an increased risk to develop invasive breast cancer.

A previous study had shown that rs2735383 may be associated with breast cancer risk in women >50 years, women with age at menarche >13 years, women with premenopausal status, women with number of abortions ≤2 and women who have breast fed, but not by age at menopause, number of pregnancies and family history^21^. Therefore, to exclude that an association of rs2735383 with breast cancer risk exists in a particular subgroup of individuals or breast cancer patients, we performed subgroup analysis according to age, age at menarche, age at menopause, menopausal status, number of full-term pregnancies, breast feeding, family history and receptor status. We did, however, not find any association between the genotype at rs2735383 and the risk of breast cancer in any of these subgroups for the recessive genetic model (Table 3). Also for the dominant and additive genetic models we found no association between the genotype at rs2735383 and breast cancer risk that would withstand multiple testing correction (Supplementary Tables S1 and S2). *NBS1* rs2735383 is thus not associated with the risk for breast cancer, either in the overall analyses or in specific subgroups.

## Discussion

The CC genotype of the common variant rs2735383 in the 3′UTR of *NBS1* has been shown to be associated with an increased cancer risk, specifically for lung and colorectal cancer (lung cancer: OR = 1.28, 95% CI = 1.21–1.46, *P* < 0.001 and colorectal cancer: OR = 1.55, 95% CI = 1.27–1.94, *P* < 10^−4^)^13^,^20^. In the current study, we evaluated the association of *NBS1* rs2735383 with breast cancer risk. We found that the CC genotype of rs2735383 did not confer an increased breast cancer risk, neither in the overall analyses nor in the subgroup analyses.

In agreement with these results, a small study by Han *et al*. consisting of 239 premenopausal breast cancer patients and 477 matched controls from the Nurses’ Health Study II showed that rs2735383 did not associate with breast cancer risk under an additive genetic model (OR = 0.92, 95% CI = 0.72–1.16, *P* = 0.469)^22^. Moreover, the study of Wu *et al*. consisting of 450 breast cancer patients and 450 cancer-free controls from the Henan Province in China also found no association with overall breast cancer risk^21^. However, after stratification according to reproductive factors, rs2735383 CC was found to be associated with an increased breast cancer risk for women >50 years, women with age at menarche >13 years, women with premenopausal status, women with number of abortions ≤2 and women who have breast fed, but not by age at menopause, number of pregnancies and family history^21^. In the current study we therefore also performed subgroup analysis by age, age at menarche, age at menopause, menopausal status, number of full-term pregnancies, breast feeding, family history and receptor status, but did not find any association between rs2735383 and risk of breast cancer in any of these subgroups that would withstand multiple testing correction. We thus could not replicate the earlier positive findings in women >50 years, women with age at menarche >13 years, premenopausal women and women who have breast fed. A possible, but not very likely, explanation for the difference in outcome between the studies may be the European versus Asian ethnicity. In the current study we chose to perform the subgroup analysis only in the European studies and not the Asian studies as this made sure that we had sufficient power in the subgroup analysis to identify smaller effects of rs2735383 on breast cancer risk. In this respect, a more plausible explanation would be that subgroup analyses, especially in a small study population (*i.e.* 450 cases and 450 controls in the study of Wu *et al*.), could have easily given rise to false positive findings. Therefore, one should be careful when reporting positive findings from multiple small subgroup comparisons and always use appropriate levels of statistical significance^23^. Unfortunately, in the study from Wu *et al*. there is no mention of multiple testing correction.

It was found that the rs2735383CC genotype significantly decreased the expression of the *NBS1* gene through either binding of microRNA-629 to the 3′-UTR of *NBS1* gene in lung cancer cells or the binding of microRNA-509-5p to the 3′-UTR of *NBS1* gene in colorectal cancer cells^19^,^20^. Since low expression of *NBS1* may reduce the efficiency of DSB repair, this way the rs2735383CC genotype likely confers an increased lung and colorectal cancer risk. According to our study, however, the rs2735383CC genotype does not confer an increased breast cancer risk. Considering that in lung and colorectal cancer cells different microRNAs appear to be downregulating *NBS1* expression, tissue specific expression of these microRNAs may likely play a role. Besides microRNA-509 and microRNA-629, the C allele at rs2735383 has also been predicted to enhance the binding of microRNA-499 and microRNA-508 to the 3′UTR of *NBS1*^21^. However, if these microRNAs are not expressed in normal breast tissue, the CC genotype of rs2735383 will not associate with breast cancer risk as *NBS1* cannot be downregulated by any of these microRNAs. At least in breast cancer cells, none of these microRNAs, except microRNA-629, are expressed at substantial levels (source: TCGA Research Network; http://cancergenome.nih.gov/). Since microRNAs are often deregulated between normal tissue and cancer tissue, this does not necessarily represent the situation in normal breast cells. Thus, further evaluation of the miRNA expression levels in normal (breast) tissue, but also their correlation with the genotype at rs2735383 should provide more insight for the tissue specificity of rs2735383 and cancer risk.

Importantly, in contrast to lung and colorectal cancer susceptibility, the results of this study do not support the presence of an association (*i.e.* OR > 1.04 for Europeans and OR > 1.11 for Asians) between the genotype at rs2735383 in the 3′UTR of *NBS1* and breast cancer susceptibility.

## Materials and Methods

### Study population

RBCS cases (N = 1,269) came from the database of the Clinical Genetics Department at Erasmus University Medical Centre in Rotterdam, representing the Southwestern part of the Netherlands. First, we selected families that presented with at least two cases of female breast cancer or at least one case of female breast cancer and one case of ovarian cancer in first- or second-degree relatives. In addition, at least one of these two cases needed to be diagnosed before the age of 60. For each selected family, the youngest breast cancer patient who had been tested for *BRCA1* and *BRCA2* was then assigned to be the index case and included in RBCS. Furthermore, breast cancer cases were also included if they were diagnosed either before the age of 40 years with unilateral breast cancer or before 50 years of age with bilateral breast cancer and did not report a family history of either breast or ovarian cancer in a first or second degree relative. All index cases and their tested relatives did not carry a *BRCA1* or *BRCA2* mutation. The median age of the RBCS cases was 44 years (range 18–92 years). RBCS controls (N = 1,159) came from the same database and geographic location as the RBCS cases and included women from cystic fibrosis families who were either spouses of individuals at risk of being carrier of a *CFTR* mutation or individuals who were tested negative for a *CFTR* mutation. The median age of the RBCS controls was 41 years (range 10–97 years).

BCAC consists of case-control studies of unrelated women^24^. For the purpose of the current analyses, only studies with participants of European and Asian ancestry were included, resulting in a total of 45 case-control studies (Supplementary Table S3). Studies with participants of African ancestry (*i.e.* two studies) were not included because power in the analyses would be low due to a relatively low MAF (*i.e.* 0.123) and small amount of cases (*i.e.* 1,046). Each study was approved by its relevant governing research ethics committee and all study participants provided written informed consent. The experimental protocol was approved by the Medical Ethical Committee of the Erasmus Medical Center Rotterdam and the study was carried out in accordance with the Code of Conduct of the Federation of Medical Scientific Societies in the Netherlands (http://www.fmwv.nl). Following genotyping on the iCOGS array^24^, quality control exclusions (described below), and analysis-specific exclusions, data from the following women were available for analysis: 47,640 patients with invasive breast cancer and 46,656 controls, totaling 94,296 BCAC participants.

### PCR-based RFLP analysis

**Figure 2:**
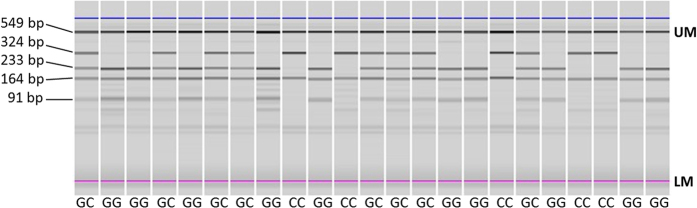
Microchip electrophoresis of the RFLP-PCR products of 23 RBCS cases. After PCR amplification of *NBS1* and *LRRC4* fragments (*i.e.* 324 and 713 bp), digestion with SfcI generated four fragments (*i.e.* 549, 233, 164 and 91 bp) for samples with rs2735383 GG genotypes, five fragments (*i.e.* 549, 324, 233, 164 and 91 bp) for samples with rs2735383 GC genotypes and three fragments (*i.e.* 549, 324 and 164 bp) for samples with rs2735383 CC genotypes. UM, upper marker; LM, lower marker.

A 324 bp fragment of the 3′UTR of *NBS1* including rs2735383 was amplified in a duplex PCR reaction together with a 713 bp fragment of the *LRRC4* gene. Primers for *NBS1* and *LRRC4* were present in the PCR reaction at a final concentration of 0.25 and 1 μM, respectively, and sequences are available in Supplementary Table S4. The amplified *LRRC4* fragment served as an internal digestion control and generated two fragments of 549 and 164 bp upon complete digestion with ScfI (New England Biolabs, Frankfurt am Main, Germany). The 324 bp amplified *NBS1* fragment was only digested when the major G allele was present at rs2735383, thereby generating two fragments of 233 and 91 bp. Thus upon successful digestion with ScfI, samples with rs2735383 GG generated four fragments, samples with rs2735383 GC generated five fragments and samples with rs2735383 CC generated three fragments (Fig. 2).

### iCOGS genotyping and imputation

Genotyping of BCAC studies was performed previously using the custom iCOGS Illumina Infinium iSelect BeadChip^24^. Briefly, DNA samples from 114,255 BCAC participants were genotyped, along with HapMap2 DNAs for European, African, and Asian populations. Raw intensity data files underwent centralized genotype calling and quality control^24^. The HapMap2 samples were used to identify women with predicted European and Asian ancestry by performing principal component (PC) analysis using a set of over 37,000 unlinked markers^25^. Nine European PCs and two Asian PCs were found to control adequately for residual population stratification in BCAC data. Samples with a low conversion rate, extreme heterozygosity, non-female sex, or one of a first-degree relative pair were excluded. Variants were excluded if they were monomorphic or had a call rate <95% (*i.e.* when MAF >0.05) or <99% (*i.e.* when MAF <0.05), deviation from HWE (*i.e. P* < 10–7), or >2% duplicate discordance.

Imputation of genotypes was performed using 1000 Genomes Project data (v3 April 2012 release) as the reference panel^26^. To improve computation efficiency we used a two-step procedure which involved pre-phasing by chromosome and by chunk using SHAPEIT software in the first step^28^ and imputation of the phased data using IMPUTE version 2 software in the second^29^. *NBS1* rs2735383 was imputed with an imputation *r*^2^ > 0.999 in both Europeans and Asians.

### Statistical analyses

The association between *NBS1* rs2735383 and invasive breast cancer risk was evaluated by logistic regression analysis providing ORs and 95% CIs. In the analyses of BCAC studies, ORs were adjusted for study, age, and PCs. In the analyses of RBCS, ethnicity was not a confounding factor thus reported ORs were unadjusted for PCs. For the European and Asian BCAC studies, we additionally performed study-specific logistic regression analysis adjusting for age and PCs, and pooled the log ORs in a fixed-effects meta-analysis. Subgroup analyses within the European BCAC studies were based on age (*i.e.* ≤50 years and >50 years), age at menarche (*i.e.* ≤13 years and >13 years), age at menopause (*i.e.* ≤50 years and >50 years), menopausal status (*i.e.* premenopausal and postmenopausal), number of full-term pregnancies (*i.e.* ≤2 and >2), breast feeding (*i.e.* no and yes), first-degree family history of breast cancer and receptor status (*i.e.* ER positive, ER negative and triple negative). Clinical and demographic characteristics of the BCAC cases are presented in Supplementary Table S5. Association between *NBS1* rs2735383 and the clinical and demographic characteristics were evaluated using a χ^2^ test. All *P*-values were two-sided and *P* < 0.05 was considered to be statistically significant after correction for multiple testing by the Bonferroni procedure. Logistic regression analyses were performed using SPSS statistics version 23 (IBM Corporation, Armonk, NY) and fixed-effects meta-analyses using Stata version 13 (StataCorp, College Station, TX).

## Additional Information

**How to cite this article**: Liu, J. *et al*. rs2735383, located at a microRNA binding site in the 3′UTR of *NBS1*, is not associated with breast cancer risk. *Sci. Rep.* **6**, 36874; doi: 10.1038/srep36874 (2016).

**Publisher’s note:** Springer Nature remains neutral with regard to jurisdictional claims in published maps and institutional affiliations.

## Supplementary Material

Supplementary Information

## Figures and Tables

**Table 1:** Association of *NBS1* rs2735383 with breast cancer risk in the RBCS study.

Genetic model	N Controls	N Cases	OR (95% CI)	*P*-value
Recessive				
GG + GC	963	1023	1	
CC	114	147	1.214 (0.936–1.574)	0.144
	1077	1170		

N, number of; MAF, minor allele frequency; OR, odds ratio; CI, confidence interval.

**Table 2:** Association of *NBS1* rs2735383 with overall breast cancer risk in the European and Asian BCAC studies.

Ethnicity	Genetic model	N Controls	N Cases	MAF Controls	MAF Cases	OR (95% CI)*	*P*-value*
European		40,0042	41,915	33.44%	33.39%		
	Recessive					1.014 (0.969–1.060)	0.556
	Dominant					1.006 (0.978–1.035)	0.684
	Additive					1.000 (0.979–1.021)	0.984
Asian		6,614	5,725	40.71%	40.58%		
	Recessive					0.998 (0.905–1.100)	0.961
	Dominant					0.995 (0.922–1.074)	0.900
	Additive					0.997 (0.946–1.050)	0.911

N, number of; MAF, minor allele frequency; OR, odds ratio; CI, confidence interval.

^*^Adjusted for age, study and principal components (PCs). In the European analyses nine PCs were added to the regression model and in the Asian analyses two PCs.

**Table 3:** Subgroup analysis of *NBS1* rs2735383 and breast cancer risk in the European BCAC studies.

Subgroup	N Controls	N Cases	MAF Controls	MAF Cases	OR (95% CI)*	*P*-value*
Age						
≤50 years	13,055	13,362	33.76%	33.41%	0.977 (0.899–1.062)	0.581
>50 years	26,987	28,553	33.28%	33.38%	1.026 (0.971–1.084)	0.356
Age at menarche						
≤13 years	14,312	13,843	33.72%	33.13%	0.984 (0.914–1.060)	0.677
>13 years	8,964	8,095	32.65%	33.63%	1.077 (0.978–1.187)	0.131
Age at menopause						
≤50 years	5,571	7,288	32,79%	33.43%	1.019 (0.906–1.146)	0.755
>50 years	3,366	4,262	33.50%	33.24%	0.993 (0.855–1.154)	0.926
Menopausal status						
Premenopausal	8,974	7,412	33.66%	33.07%	0.981 (0.887–1.085)	0.715
Postmenopausal	19,648	17,353	33.39%	33.56%	1.007 (0.943–1.075)	0.844
Number of full-term pregnancies						
≤2	21,008	19,722	33.53%	33.20%	1.004 (0.942–1.071)	0.893
>2	8,258	7,327	33.26%	33.44%	1.032 (0.931–1.144)	0.549
Breast feeding						
No	6,849	6,805	33.36%	33.25%	0.988 (0.884–1.104)	0.828
Yes	11,947	12,709	33.68%	33.28%	0.978 (0.903–1.060)	0.594
Family history						
1^st^ degree relative with BC	23,648	4,119	33.21%	32.68%	0.990 (0.884–1.108)	0.859
Receptor status						
ER positive	39,699	25,959	33.47%	33.40%	1.021 (0.970–1.075)	0.427
ER negative	39,618	6,774	33.42%	32.90%	0.991 (0.908–1.082)	0.846
Triple negative	30,696	2,712	33.10%	32.04%	0.980 (0.847–1.134)	0.788

N, number; MAF, minor allele frequency; OR, odds ratio; CI, confidence interval; ER, estrogen receptor; BC, breast cancer.

^*^Recessive genetic model adjusted for age, study and nine principal components.
