# Supplementary material for: rs2735383, located at a microRNA binding site in the 3’UTR of NBS1, is not associated with breast cancer risk
Source: Sci Rep. 2016 Nov 15;6:36874. doi: 10.1038/srep36874 (PMC5109293; doi:10.1038/srep36874)
Supplement: Supplementary Information [file srep36874-s1.pdf]

rs2735383, located at a microRNA binding site in the 3’UTR of *NBS1*, is not associated with breast cancer risk

Jingjing Liu, Ivona Lončar, J. Margriet Collée, Manjeet K. Bolla, Joe Dennis, Kyriaki Michailidou, Qin Wang, Irene L. Andrulis, Monica Barile, Matthias W. Beckmann, Sabine Behrens, Javier Benitez, Carl Blomqvist, Bram Boeckx, Natalia V. Bogdanova, Stig E. Bojesen, Hiltrud Brauch, Paul Brennan, Hermann Brenner, Annegien Broeks, Barbara Burwinkel, Jenny Chang-Claude, Shou-Tung Chen, Georgia Chenevix-Trench, Ching Y. Cheng, Ji-Yeob Choi, Fergus J. Couch, Angela Cox, Simon S. Cross, Katarina Cuk, Kamila Czene, Thilo Dörk, Isabel dos-Santos-Silva, Peter A. Fasching, Jonine Figueroa, Henrik Flyger, Montserrat García-Closas, Graham G. Giles, Gord Glendon, Mark S. Goldberg, Anna González-Neira, Pascal Guénel, Christopher A. Haiman, Ute Hamann, Steven N. Hart, Mikael Hartman, Sigrid Hatse, John L. Hopper, Hidemi Ito, Anna Jakubowska, Maria Kabisch, Daehee Kang, Veli-Matti Kosma, Vessela N. Kristensen, Loic Le Marchand, Eunjung Lee, Jingmei Li, Artitaya Lophatananon, Jan Lubinski, Arto Mannermaa, Keitaro Matsuo, Roger L. Milne, NBCS Collaborators, Susan L. Neuhausen, Heli Nevanlinna, Nick Orr, Jose I.A. Perez, Julian Peto, Thomas C. Putti, Katri Pylkäs, Paolo Radice, Suleeporn Sangrajrang, Elinor J. Sawyer, Marjanka K. Schmidt, Andreas Schneeweiss, Chen-Yang Shen, Martha J. Shrubsole, Xiao-Ou Shu, Jacques Simard, Melissa C. Southey, Anthony Swerdlow, Soo H. Teo, Daniel C. Tessier, Somchai Thanasitthichai, Ian Tomlinson, Diana Torres, Thérèse Truong, Chiu-Chen Tseng, Celine Vachon, Robert Winqvist, Anna H. Wu, Drakoulis Yannoukakos, Wei Zheng, Per Hall, Alison M. Dunning, Douglas F. Easton, Maartje J. Hooning, Ans M.W. van den Ouweland, John W.M. Martens, Antoinette Hollestelle.

Supplementary Data

Supplementary Table S1. Subgroup analysis of *NBS1* rs2735383 under a dominant genetic model and breast cancer risk in the European BCAC studies.

Subgroup	N Controls	N Cases	MAF Controls	MAF Cases	OR (95% CI)*	*P*-value*
Age						
≤ 50 years	13,055	13,362	33.76%	33.41%	0.995 (0.944-1.050)	0.995
> 50 years	26,987	28,553	33.28%	33.38%	1.006 (0.971-1.042)	0.742
Age at menarche						
≤ 13 years	14,312	13,843	33.72%	33.13%	0.961 (0.916-1.008)	0.106
> 13 years	8,964	8,095	32.65%	33.63%	1.065 (1.002-1.132)	0.044
Age at menopause						
≤ 50 years	5,571	7,288	32,79%	33.43%	1.036 (0.962-1.115)	0.349
> 50 years	3,366	4,262	33.50%	33.24%	0.977 (0.888-1.074)	0.628
Menopausal status						
Premenopausal	8,974	7,412	33.66%	33.07%	0.969 (0.908-1.033)	0.332
Postmenopausal	19,648	17,353	33.39%	33.56%	1.011 (0.970-1.054)	0.608
Number of full-term pregnancies						
≤ 2	21,008	19,722	33.53%	33.20%	0.999 (0.959-1.041)	0.974
> 2	8,258	7,327	33.26%	33.44%	1.017 (0.952-1.086)	0.624
Breast feeding						
No	6,849	6,805	33.36%	33.25%	0.991 (0.923-1.064)	0.810
Yes	11,947	12,709	33.68%	33.28%	0.988 (0.939-1.041)	0.657
Family history						
1st degree relative with BC	23,648	4,119	33.21%	32.68%	0.999 (0.931-1.073)	0.985
Receptor status						
ER-positive	39,699	25,959	33.47%	33.40%	1.006 (0.973-1.039)	0.740
ER-negative	39,618	6,774	33.42%	32.90%	0.997 (0.943-1.053)	0.904
Triple negative	30,696	2,712	33.10%	32.04%	0.987 (0.901-1.080)	0.770

N, number; MAF, minor allele frequency; OR, odds ratio; CI, confidence interval; ER, estrogen receptor; BC, breast cancer.
*Adjusted for age, study and nine principal components.

Supplementary Table S2. Subgroup analysis of *NBS1* rs2735383 under an additive genetic model and breast cancer risk in the European BCAC studies.

Subgroup	N Controls	N Cases	MAF Controls	MAF Cases	OR (95% CI)*	*P*-value*
Age						
≤ 50 years	13,055	13,362	33.76%	33.41%	0.992 (0.954-1.032)	0.699
> 50 years	26,987	28,553	33.28%	33.38%	1.009 (0.983-1.036)	0.495
Age at menarche						
≤ 13 years	14,312	13,843	33.72%	33.13%	0.975 (0.941-1.010)	0.163
> 13 years	8,964	8,095	32.65%	33.63%	1.053 (1.006-1.102)	0.027
Age at menopause						
≤ 50 years	5,571	7,288	32,79%	33.43%	1.024 (0.969-1.082)	0.398
> 50 years	3,366	4,262	33.50%	33.24%	0.986 (0.918-1.058)	0.686
Menopausal status						
Premenopausal	8,974	7,412	33.66%	33.07%	0.979 (0.933-1.026)	0.374
Postmenopausal	19,648	17,353	33.39%	33.56%	1.008 (0.977-1.039)	0.635
Number of full-term pregnancies						
≤ 2	21,008	19,722	33.53%	33.20%	1.001 (0.971-1.031)	0.969
> 2	8,258	7,327	33.26%	33.44%	1.016 (0.968-1.067)	0.518
Breast feeding						
No	6,849	6,805	33.36%	33.25%	0.992 (0.942-1.046)	0.778
Yes	11,947	12,709	33.68%	33.28%	0.989 (0.952-1.027)	0.560
Family history						
1st degree relative with BC	23,648	4,119	33.21%	32.68%	0.997 (0.946-1.052)	0.923
Receptor status						
ER-positive	39,699	25,959	33.47%	33.40%	1.008 (0.983-1.033)	0.533
ER-negative	39,618	6,774	33.42%	32.90%	0.996 (0.956-1.038)	0.856
Triple negative	30,696	2,712	33.10%	32.04%	0.988 (0.923-1.057)	0.731

N, number; MAF, minor allele frequency; OR, odds ratio; CI, confidence interval; ER, estrogen receptor; BC, breast cancer.
*Adjusted for age, study and nine principal components.

Supplementary Table S3. Participating Breast Cancer Association Consortium (BCAC) studies.

A. European studies

Study	Abbreviation	Country	Study design	Design category	Case definition	Control definition	Participation rates reported by Investigator	Selected familial cases	Controls	Cases with invasive BC	References
Australian Breast Cancer Family Study	ABCFS	Australia	Population-based case-control study	Population-based	All cases diagnosed < age 40 plus a random sample of those diagnosed ages 40-59 from cancer registries in Victoria and New South Wales, plus a limited number diagnosed aged 60-69; cases living in Melbourne recruited from 1992-99 and in Sydney from 1993-98.	Identified from the electoral rolls in Melbourne from 1992-98 and Sydney from 1993-99. Frequency matched to cases by age in 5 year categories.	75% of cases and 68% of controls completed questionnaires, 71% of cases and 55% of controls provided a blood sample	No	537	736	Dite, G.S. et al. Familial risks, early-onset breast cancer, and BRCA1 and BRCA2 germline mutations. J. Natl. Cancer Inst., 95, 448-57 (2003).
Amsterdam Breast Cancer Study	ABCS	Netherlands	Hospital-based consecutive cases; population-based controls	Mixed	Breast cancer patients diagnosed before age 50 in 1995-2011 at the Netherlands Cancer Insitute - Antoni van Leeuwenhoek hospital (NKI-AVL).	Population-based cohort of women recruited through the Sanquin blood bank, all ages.	85% of cases and ~50% of controls for DNA	Subset	1556	1878	M. K. Schmidt, et al. Breast Cancer Survival and Tumor Characteristics in Premenopausal Women Carrying the CHEK2*1100delC Germline Mutation, J.C.O., 06, 3024 (2006)
Bavarian Breast Cancer Cases and Controls	BBCC	Germany	Hospital-based cases; population based controls	Mixed	Consecutive, unselected cases with invasive breast cancer recruited at the University Breast Centre, Franconia in Northern Bavaria during 1999-2013.	Healthy women with no diagnosis of cancer aged 55 or older. Invited by a newspaper advertisement in Northern Bavaria, and recruited during 1999-2013.	95% of cases and 99% of controls provided a blood sample and an epidemiological questionnaire.	No	458	554	**1)** Fasching PA, et al. Single nucleotide polymorphisms of the aromatase gene (CYP19A1), HER2/neu status, and prognosis in breast cancer patients. Breast Cancer Res Treat. DOI 10.1007/s10549-007-9822-2 (2007). **2)** Schrauder M, et al. Single nucleotide polymorphism D1853N of the ATM gene may alter the risk for breast cancer. J. Cancer Res. Clin. Oncol.,134, 873-82 (2008).
British Breast Cancer Study	BBCS	UK	Cancer registry and National Cancer Research network (NCRN) based cases; population-based controls	Mixed	1) English & Scottish Cancer Registries: all breast cancer cases who developed a first primary before age 65 in 1971 or later and who subsequently developed a second primary cancer. 2) Unilateral breast cancer cases diagnosed before age 70 in 1971 or later.	1) A friend, sister-in-law, daughter-in-law or other non-blood relative of cases. Recruitment of cases and controls began in January 2001.	1) 68% of cases & 76% of controls provided a blood sample. 2) 82% provided a blood sample.	No	1326	1423	**1)** Johnson, N. et al. Interaction between CHEK2*1100delC and other low-penetrance breast-cancer susceptibility genes: a familial study. Lancet, 366, 1554-7 (2005). **2)** Fletcher, O. et al. Inconsistent association between the STK15 F31I genetic polymorphism and breast cancer risk. J. Natl. Cancer. Inst., 98, 1014-8 (2006).
Breast Cancer in Galway Genetic Study	BIGGS	Ireland	Hospital-based cases; population based controls	Mixed	Unselected cases recruited from West of Ireland since 2001. Cases were recruited from University College Hospital Galway and surrounding hospitals	Women > 60 years with no personal history of any cancer and no family History of breast or ovarian cancer were identified from retirement groups in the West of Ireland (same catchment area as cases) during the period 2001-2008.	Not recorded	No	49	783	**1)** Colleran G, McInerney N, Rowan A, Barclay E, Jones AM, Curran C, Miller N, Kerin M, Tomlinson I, Sawyer E. The TGFBR1*6A/9A polymorphism is not associated with differential risk of breast cancer. Breast Cancer Res Treat. 2009 Apr 24. **2)** Niall Mcinerney, Gabrielle Colleran, Andrew Rowan, Axel Walther, Ella Barclay, Sarah Spain Angela M. Jones Stephen Tuohy, Catherine Curran, Nicola Miller, Michael Kerin, Ian Tomlinson, Elinor J. Sawyer. Low penetrance breast cancer predisposition SNPs are site specific.
Breast Cancer Study of the University of Heidelberg	BSUCH	Germany	Hospital-based cases; healthy blood donator controls	Mixed	Cases diagnosed with breast cancer/breast cancer metastasis in 2008-2011 at the University Women`s Clinic Heidelberg.	Healthy, unrelated, ethnically matched female blood donors recruited in 2007, 2009 & 2012 by German Red Cross Blood Service of Baden-Württemberg-Hessen, Institute of Transfusion Medicine & Immunology, Mannheim.	Cases: 82% Controls:Offers of blood donation from approximately 5% of donors were refused due to various reasons	No	954	814	Yang,R. et al. Genetic variants within miR-126 and miR-335 are not associated with breast cancer risk. Breast Cancer Res Treat 127, 549-554 (2011).
CECILE Breast Cancer Study	CECILE	France	Population-based case-control study	Population-based	All incident cases of breast cancer diagnosed in 2005-2007 among women <75 years of age and residing in Ille-et-Vilaine or Côte d'Or. Cases were recruited from the main cancer treatment center (Centre Eugène-Marquis in Rennes and Centre Georges-François-Leclerc in Dijon) and from private or public hospitals in each area.	General population control women residing in the same geographic areas frequency-matched to the cases by 5-year age groups. Controls were recruited in 2005-2007 by phone using a random digit dialing procedure and predefined numbers by socioeconomic status to control for possible selection bias.	In-person interviewes completed for 77% of cases, and 74% of controls. Among interviewed subjects, 85% of cases and 78% of controls provided a blood sample, and 12 % of cases and 19% of controls provided DNA from buccal cell samples	No	997	900	Menegaux F, Truong T, Anger A, Cordina-Duverger E, Lamkarkach F, Arveux P, Kerbrat P, Fevotte J, Guenel P: Night work and breast cancer: a population-based case-control study in France (the CECILE study). Int J Cancer 2013, 132(4):924-931.
Copenhagen General Population Study	CGPS	Denmark	Population-based case-control study	Mixed	Consecutive, incident cases from 1 hospital with centralized care for a population of 400,000 women from 2001 to the present.	Community controls residing in the same region as cases and with no history of breast cancer were identified from the Copenhagen General Population Study recruited 2003-2007. All controls were known to still be breast cancer-free at the end of 2007.	96% of cases and 56% of controls were interviewed and provided a blood sample.	No	4519	2811	Weischer,M., Bojesen,S.E., Tybjaerg-Hansen,A., Axelsson,C.K., & Nordestgaard,B.G. Increased risk of breast cancer associated with CHEK2*1100delC. J Clin Oncol 25, 57-63 (2007).
Spanish National Cancer Centre Breast Cancer Study	CNIO-BCS	Spain	Case-control study	Mixed	Two groups of cases:1) 574 consecutive breast cancer patients, unselected for family history, from 3 public hospitals, 2 in Madrid and one in Oviedo, from 2000 to 2005. 2) 291 cases with at least one first degree relative also affected with breast cancer, recruited through the CNIO family cancer clinic in Madrid from 2000 to 2004.	Women attending the Menopause Research Centre between 2000 and 2004 and female members of the College of Lawyers attending a free, targeted medical check-up in 2005, all free of breast cancer and all in Madrid	Not recorded.	Subset	834	704	Milne, RL et al. ERCC4 associated with breast cancer risk: a two stage case-control study using high throughput genotyping. Cancer Res., 66, 9420-7 (2006)

California Teachers Study	CTS	USA	Prospective cohort study: nested case-control	Prospective cohort	This is a nested case-control study conducted within a cohort of California teachers(113,590) who were under age 80 years at baseline, had no prior history of invasive or in situ breast cancer. Cases are women newly diagnosed with a histologically confirmed invasive primary adenocarcinoma of the breast at age 80 years or younger from 1998 to 2008.	Controls are a probability sample of at-risk cohort members, frequency matched to cases on age at baseline (5-year age groups), self-reported race/ethnicity (white, African American, Latina, Asian, other), and broad geographic region within California. Controls were recruited during 1998 to 2008 and selected without replacement, using an assigned reference date.		No	44	51	Bernstein L, Allen M, Anton-Culver H, Deapen D, Horn-Ross PL, Peel D, Pinder R, Reynolds P, Sullivan-Halley J, West D et al. High breast cancer incidence rates among California teachers: results from the California Teachers Study (United States). Cancer Causes Control 2002, 13(7):625-635.
ESTHER Breast Cancer Study	ESTHER	Germany	Population-based case-control study	Population-based	Statewide recruitment of breast cancer cases in all hospitals in Saarland/Germany in 2001-2003.	Statewide recruitment of participants of a routine health check-up in Saarland/Germany in 2000-2002. A stratified random sample, matched to the cases by five year age groups, was selected as controls.	Not recorded	Subset	502	471	Widschwendter M, Apostolidou S, Raum E, Rothenbacher D, Fiegl H, Menon U, Stegmaier C, Jacobs IJ, Brenner H. Epigenotyoing in peripheral blood cell DNA and breast cancer risk: a proof of principle study. PloS ONE 2008;3:e2656.
Gene Environment Interaction and Breast Cancer in Germany	GENICA	Germany	Population-based case-control study	Population-based	Incident breast cancer cases enrolled between 2000 and 2004 from the Greater Bonn area (by of the hospitals within the study region); all enrolled within 6 months of diagnosis.	Selected from population registries from 31 communities in the greater Bonn area; matched to cases in 5-year age classes between 2001 and 2004	Response rate 88% for cases and 67% for controls. Of these, DNA samples are available for 89% and 90% respectively.	No	427	465	**1)** Pesch, B. et al. Factors modifying the association between hormone –replacement therapy and breast cancer risk. Eur. J. Epidemiol., 20:699-711 (2005). **2)** Justenhoven,C. et al. The CYP1B1_1358_GG genotype is associated with estrogen receptor-negative breast cancer. Breast Cancer Res Treat. 111, 171-177 (2008).
Helsinki Breast Cancer Study	HEBCS	Finland	Hospital-based case control study, plus additional familial cases	Mixed	(1) Consecutive cases (883) from the Department of Oncology, Helsinki University Central Hospital 1997-8 and 2000, (2) Consecutive cases (986) from the Department of Surgery, Helsinki University Central Hospital 2001 – 2004, (3) Familial breast cancer patients (536) from the Helsinki University Central Hospital, Departments of Oncology and Clinical Genetics (1995-)	Healthy females from the same geographical region in Southern Finland in 2003.	(1) 79% of all cases for the 1. consecutive series, (2) 87% of all cases for the 2. consecutive series 87% , (3) about 90% of the familial cases. Controls (100%).	Subset	1058	1512	**1)** Syrjakoski, K. et al. Population-based study of BRCA1 and BRCA2 mutations in 1035 unselected Finnish breast cancer patients. J. Natl. Cancer Inst., 92, 1529-31 (2000). **2)** Kilpivaara, O. et al. Correlation of CHEK2 protein expression and c.1100delC mutation status with tumor characteristics among unselected breast cancer patients. Int J. Cancer, 113, 575-80 (2005).**3)** Fagerholm,R. et al. NAD(P)H:quinone oxidoreductase 1 NQO1*2 genotype (P187S) is a strong prognostic and predictive factor in breast cancer. Nat Genet 40, 844-853 (2008).
Hannover-Minsk Breast Cancer Study	HMBCS	Belarus	Hospital-based cases; population based controls	Mixed	Ascertainment at the Byelorussian Institute for Oncology and Medical Radiology Aleksandrov N.N. in Minsk or at one of 5 regional oncology centers in Gomel, Mogilev, Grodno, Brest or Vitebsk through the years 2002-2008.	Controls from the same population aged 18-72 years. Healthy (without personally history of cancer) female probunds recruited from the same geographical regions as cases during the years 2002-2008. About 75% of controls were women invited for general medical examination at five regional gynecology clinics (in Gomel, Mogilev, Grodno, Brest or Vitebsk) and cancer-free volunteers ascertained at the Institute for Inherited Diseases in Minsk; 20% were cancer-free female blood bank donors recruited at Republic Blood Bank, Minsk, Belarus; finally 5% of controls were healthy cancer-free relatives of some breast cancer patients.	More than 60% for cases and more than 80% for controls. DNA available from all of the participants.	No	130	686	Bogdanova,N. et al. A nonsense mutation (E1978X) in the ATM gene is associated with breast cancer. Breast Cancer Res Treat 118, 207-211 (2009).
Kuopio Breast Cancer Project	KBCP	Finland	Population-based prospective clinical cohort	Population-based	Women seen at Kuopio University Hospital between 1990 and 1995 because of breast lump, mammographic abnormality, or other breast symptom who were found to have breast cancer	Age and long-term area-of-residence matched controls selected from the National Population Register and interviewed in parallel with the cases	Cases: 98% of those contacted; which is 86% of those potentially eligible. Controls were selected individually for each case (response rate not available)	No	250	410	**1)** Hartikainen, J.M. et al. An autosome-wide scan for linkage disequilibrium-based association in sporadic breast cancer cases in eastern Finland: three candidate regions found. Cancer Epidemiol. Biomarkers Prev., 14, 75-80 (2005). **2)** Hartikainen,J.M. et al. Refinement of the 22q12-q13 breast cancer-associated region: evidence of TMPRSS6 as a candidate gene in an eastern Finnish population. Clin Cancer Res 12, 1454-1462 (2006).
Kathleen Cuningham Foundation Consortium for research into Familial Breast Cancer/Australian Ovarian Cancer Study	KConFab/AOCS	Australia and New Zeland	Clinic-based recruitment of familial breast cancer patients (cases); population-based case-control study of ovarian cancer (controls only)	Mixed	Cases were from multiple-case breast and breast-ovarian families recruited though family cancer clinics from across Australia and New Zealand from 1998 to the present. Cases were selected for inclusion in BCAC studies if (i) family was negative for mutations in BRCA1 and BRCA2 (ii) case was the index for the family, defined as youngest breast cancer affected family member.	Female controls were ascertained by the Australian Ovarian Cancer Study identified from the electoral rolls from all over Australia from 2002-2006.	78% family members approached agreed to participate. Of those, 97% provided a blood sample and 96% provided questionnaire data	Yes	876	359	**1)** Mann, G.J. et al. Analysis of cancer risk and BRCA1 and BRCA2 mutation prevalence in the kConFab familial breast cancer resource. Breast Cancer Res., 8, R12 (2006). **2)** Beesley, J et al. Association between single nucleotide polymorphisms in hormone metabolism and DNA repair genes and epithelial ovarian cancer: Results from two Australian studies and an additional validation set. Cancer Epidemiol. Biomarkers Prev., 12, 2257-65 (2007).
Leuven Multidisciplinary Breast Centre	LMBC	Belgium	Hospital-based case control study	Mixed	All patients diagnosed with breast cancer and seen in the Multidisciplinary Breast Center in Leuven (Gashuisberg) since June 2007 plus retrospective collection of cases diagnosed since 2000	Healthy controls (blood donors) collected at the Red Cross and located in Gasthuisberg hospital (Oct-2007-March 2008)	High participation rate. At least 90% of patients and controls participated to studies. Few people are unwilling.	No	1386	2491	**1)** Neven P, Brouckaert O, Van Belle V, Vanden Bempt I, Hendrickx W, Cho H, Deraedt K, Van Calster B, Van Huffel S, Moerman P, Amant F, Leunen K, Smeets A, Wildiers H, Paridaens R, Vergote I, Christiaens MR. In early-stage breast cancer, the estrogen receptor interacts with correlation between human epidermal growth factor receptor 2 status and age at diagnosis, tumor grade, and lymph node involvement. J Clin Oncol. 2008 Apr 1;26(10):1769-71. **2)**
Mammary Carcinoma Risk Factor Investigation	MARIE	Germany	Population-based case-control study	Population-based	Incident cases diagnosed from 2001-2005 in the study region Hamburg in Northern Germany, and from 2002-2005 in the study region Rhein-Neckar-Karlsruhe in Southern Germany.	2 controls per case were randomly drawn from population registries and frequency matched by birth year and study region to the case. Controls were recruited from 2002 to 2006.	64.1% of cases & 43.4% of controls participated (rates for people with QX)	No	1778	1655	Flesch-Janys, D et al.Risk of different histological types of postmenopausal breast cancer by type and regimen of menopausal hormone therapy Int J Cancer. 2008 Aug 15;123(4):933-41.

Milan Breast Cancer Study Group	MBCSG	Italy	Clinic-based recruitment of familial/early onset breast cancer patients (cases); population-based controls	Mixed	Familial and/or early onset breast cancer patients (aged 22-87) negative for mutations in BRCA genes, ascertained in two large cancer centres in Milan from 2000 to date.	Healthy blood donors aged 18-71 years, retruited at two blood centres in Milan from 2004 (centre 1) and 2007 (centre 2) to date	>99%	Yes (ca. 90%)	400	189	**1)** De Vecchi et al. Evidences for association of the CASP8 –652 6N del promoter polymorphism with age at diagnosis in familial breast cancer cases (letter). Breast Cancer Res Treat 113:607-8, 2009. **2)** Catucci et al. Letter to the editor: SNPs in ultraconserved elements and familial breast cancer risk. Carcinogenesis 30:544–545, 2009.
Mayo Clinic Breast Cancer Study	MCBCS	USA	Hospital-based case control study	Mixed	Incident cases residing in 6 states (MN, WI, IA, IL, ND, SD) seen at the Mayo Clinic in Rochester, MN from 2002-5	Women without cancer presenting for general medical examination at the Mayo Clinic. Controls were recruited concurrently with cases and were frequency matched to cases on age, ethnicity and county/state	68% for cases, 77% for controls were interviewed and provided a blood sample	No	1923	1535	Olson, JE. et al. A comprehensive examination of CYP19 variation and breast density. Cancer Epidemiol. Biomarkers Prev. 16, 623-5 (2007)
Melbourne Collaborative Cohort Study	MCCS	Australia	Prospective cohort study: nested case-control study	Prospective cohort	Incident cases diagnosed between baseline (1990-1994) and last follow-up (2012) among the 24469 women participating in the cohort.	For each case a control was randomly selected from women from the cohort who did not develop breast cancer before the age at diagnosis of the case and matched the case on year of birth and country of birth.	1	No	511	614	Giles GG. Et al. The Melbourne Collaborative Cohort Study. IARC Sci. Publ., 156, 69-70 (2002)
Multiethnic Cohort	MEC	USA	Prospective cohort study: nested case-control	Prospective cohort	Incident cases identified from SEER cancer registries in Los Angeles County & State registries in California & Hawaii, USA from 1993-2002. Grouped by self-reported ethnicity.	Women without cancer from the same States, recruited concurrently with cases & frequency matched to cases by age at blood-draw & self-reported ethnicity.	>60% for both cases & controls	No	741	703	Kolonel, L. N. et al. A multi-ethnic cohort in Hawaii and Los Angeles; Baseline characteristics. Am. J. Epidemiol., 151, 346-357 (2000)
Montreal Gene-Environment Breast Cancer Study	MTLGEBCS	Canada	Population-based case-control study	Population-based	All cases are postmenopausal women (47-75 years) living in Montreal with a primary invasive breast cancer and with no previous occurrence of any type of cancer. All cases were identified from 2007 to 2010 in 15 of 16 Montreal hospitals that treat breast cancer.	All controls are postmenopausal women (47-75 years) living in Montreal with no personal history of cancer. All controls were identified using the Quebec provincial electoral list from 2007-2010. The electoral list has close to 100% coverage of Canadian citizens living in the Province.	57% for the cases and 41% for the controls (estimates for controls are difficult to estimate)	No	360	409	Unpublished
Norwegian Breast Cancer Study	NBCS	Norway	Hospital-based case control study	Mixed	Incidence cases from three different hospitals: 1) Cases (114) mean age 64 (28-92) at Ullevål Univ. Hospital 1990-94, 2) cases (182) mean age 59 (26-75) referred to Norwegian Radium Hospital 1975-1986, 3) cases (124), mean age 56 (29-82)) with stage I or II disease, in the Oslo micro-metastases study at Norwegian Radium Hospital between 1995-1998, 4) Breast cancer cases referred to the Norwegian hospitals Akershus University Hospital in Lørenskog, Ullevaal university hospital in Oslo and Rikshospitalet-Radiumhospitalet in Oslo from 2007-2010. Mean age is 63 years. Consecutive series. 5) Breast cancer cases referred to the Norwegian Radium Hospital hospitalet 2010-2013. Neoadjuvantly treated with Avastin (Bevacizumab). 6) Consecutive series of Breast cancer incidents referred to Akershus university hospital 2004-2014.	Control subjects were healthy women, age 55-71, residing in Tromsø (440), and Bergen (109) attending the Norwegian Breast Cancer Screening Program. Healthy tissue from mammoplastic reduction surgery at a private clinic in Oslo.	80-82% cases and 70% controls	No	214	828	**1)** Aure et al. Genome Med. 2015 Feb 2;7(1):21. **2)** Fleischer et al. 2014 Genome Biol. 2014;15(8):435. **3)** Fleischer et al. 2014 Int J Cancer. 2014 Jun 1;134(11):2615-25. **4)** Quigley et al. 2014 Mol Oncol. 2014 Mar;8(2):273-84.
Nashville Breast Health Study	NBHS	USA	Population-based case-control study	Population-based	Through a rapid case-ascertainment system, we identified newly-diagnosed breast cancer cases through the Tennessee State Cancer Registry and five major hospitals in the city that provide medical care for breast cancer patients. Eligible cases were women diagnosed with invasive breast cancer or ductal carcinoma in situ, who were between the ages of 25 and 75, had no prior history of cancer other than non-melanoma skin cancer, had a resident telephone, spoke English, and who were able to provide consent to the study. Recruitment period was from 2001 to 2011. The recruitment for European Americans ended in 2008.	Controls were identified via random digit dialing (RDD) of households in the same geographic area as cases during 2001-2011. Eligibility criteria for controls were the same as cases with the exception that controls did not have a prior cancer diagnosis other than simple skin cancer. Controls were frequency matched to cases on 5-year age group, race, and county of residence.	58% for cases and 48% for controls.	No	118	125	Zheng W, Long J, Gao YT, Li C, Zheng Y, Xiang YB, Wen W, Levy S, Deming SL, Haines JL, Gu K, Fair AM, Cai Q, Lu W, Shu XO. Genome-wide association study identifies a new breast cancer susceptibility locus at 6q25.1. Nature Genetics 41(3):324-8, 2009. PMCI.
Oulu Breast Cancer Study	OBCS	Finland	Hospital-based case control study	Mixed	Consecutive incident cases diagnosed at the Oulu University Hospital between 2000 and 2004.	Healthy, consecutive, anonymous, female Finnish Red-Cross blood donors recruited in 2002 from the same geographical region in Northern Finland.	All of the asked controls, and 71% of all cases treated at the Oulu University Hospital, Department of Oncology during the collection period.	No	414	500	Erkko,H. et al. A recurrent mutation in PALB2 in Finnish cancer families. Nature 446, 316-319 (2007).

Ontario Familial Breast Cancer Registry	OFBCR	Canada	Population-based familial case-control study	Mixed	Cases diagnosed between 1 Jan 1996-31 Dec 1998 were identified from the Ontario Cancer Registry which registers >97% of all cases residing in the province at the time of diagnosis. All women with invasive breast cancer aged 20–54 years who met the OFBCR definition for high genetic risk (family history of specific cancers particularly breast and ovarian, early onset disease, Ashkenazi ethnicity or a diagnosis of multiple breast cancer) were asked to participate by completing risk factor questionnaires and providing a blood sample. A 25% random sample of individuals in this age category who did not meet the OFBCR definition, 35% of those aged 55–69 at high risk and 8.75% aged 55–69 at low risk were also asked to participate. Individuals diagnosed in 2001 and 2002 were also included if they met high -risk criteria.	Unrelated, unaffected population controls were recruited between 2003-2005 by calling randomly selected residential telephone numbers throughout the same geographical region. Eligible controls were women with no history of breast cancer and were frequency-matched by 5-year age group to the expected age distribution of cases. Approximately, 65% of identified eligible women returned questionnaires, and 63% of these donated a blood specimen.	Cases: consent to contact patients was 92%, response to initial family history questionnaire was 65%, response to risk factor questionnaires was 73% of all eligible, and donation of a blood sample was 63% of all eligible. Less than 2% died before initial contact. Controls: approximately, 65% of identified eligible women returned questionnaires, and 63% of these donated a blood specimen.	Subset	496	957	John,E.M. et al. The Breast Cancer Family Registry: an infrastructure for cooperative multinational, interdisciplinary and translational studies of the genetic epidemiology of breast cancer. Breast Cancer Res 6, R375-R389 (2004).
NCI Polish Breast Cancer Study	PBCS	Poland	Population-based case-control study	Population-based	Incident cases from 2000-2003 identified through a rapid identification system in participating hospitals covering ~ 90% of all eligible cases, and cancer registries in Warsaw and Łódź covering 100% of all eligible cases.	Randomly selected from population lists of all residents of Poland, stratified and frequency matched to cases by case city and age in 5 year categories. Recruited 2000-2003.	79% of eligible cases and 69% of eligible controls agreed to personal interview; 84% of interviewed cases and 94% of interviewed controls provided a DNA sample	No	424	519	Garcia-Closas, M. et al. Polymorphisms in DNA double-strand break repair genes and risk of breast cancer: two population-based studies in USA and Poland, and meta-analyses. Hum. Genet, 119, 376-88 (2006).
Karolinska Mammography Project for Risk Prediction of Breast Cancer - Case-Control Study	pKARMA	Sweden	Case-control study	Mixed	Incident cases from Jan 2001 – Dec 2008 from the Stockholm/Gotland area. Identified through the Stockholm breast cancer registry.	Unmatched participants of the KARMA mammography screening study recruited between 2010 and 2011 from Helsingborg and Stockholm.	60% for the cases. Unknown for KARMA controls.	No	5505	4497	Unpublished
Singapore and Sweden Breast Cancer Study	SASBAC	Sweden	Population-based case-control study	Population-based	Incident cases from October 1993 to March 1995 identified via the 6 regional cancer registries in Sweden, to which reporting is mandatory.	Controls were randomly selected from the total population registry in 5-year age groups to match the expected age-frequency distribution among cases. Patients and controls were recruited from Oct 1993 through April 1995.	84% of cases & 82% of controls questionnaire, 87% & 74% of those donated DNA (overall 73% & 61% respectively).	No	1378	1163	Wedren, S. et al. Oestrogen receptor alpha gene haplotype and postmenopausal breast cancer risk: a case control study. Breast Cancer Res., 6, R437-49 (2004).
Sheffield Breast Cancer Study	SBCS	UK	Hospital-based case control study	Mixed	Women with pathologically confirmed breast cancer recruited from surgical outpatient clinics at the Royal Hallamshire Hospital, Sheffield, 1998 – 2005; cases are a mixture of prevalent and incident disease	Unselected women attending the Sheffield Mammography Screening Service between Sep 2000 - Aug 2004, if their mammograms showed no evidence of a breast lesion	Not recorded	No	848	749	**1)** MacPherson,G. et al. Association of a common variant of the CASP8 gene with reduced risk of breast cancer. Journal of the National Cancer Institute 96, 1866-1869 (2004). **2)** Rafii,S. et al. A potential role for the XRCC2 R188H polymorphic site in DNA-damage repair and breast cancer. Human Molecular Genetics 11, 1433-1438 (2002).
Study of Epidemiology and Risk factors in Cancer Heredity	SEARCH	UK	Population-based case-control study	Mixed	2 groups of cases identified through East Anglian Cancer Registry; 1) prevalent cases diagnosed 1991-1996 under 55 years of age at diagnosis, recruited 1996-2002; 2) incident cases diagnosed since 1996 under 70 years of age at diagnosis, recruited 1996-present.	Two groups of controls: (1) selected from the EPIC-Norfolk cohort study of 25,000 individuals age 45-74 recruited between 1992 and1994, based in the same geographic region as cases; (2) selected from GP practices from March 2003 to present, frequency matched to cases by age and geographic region	64% of eligible cases and 41% of invited controls provided a blood sample	No	8063	9087	Lesueur, F. et al. Allelic association of the human homologue of the mouse modifier Ptprj with breast cancer. Hum. Mol. Genet., 14, 2349-56 (2005).
Städtisches Klinikum Karlsruhe Deutsches Krebsforschungszentrum Study	SKKDKFZS	Germany	Hospital-based breast cancer cohort	Patient cohort	Women diagnosed with primary *in situ* or invasive breast cancer at the Städtisches Klinikum Karlsruhe from March 1993 to July 2005.	No controls.	Not recorded	No	29	131	Stevens, K.N. et al. 9p13.1 is a triple-negative-specific breast cancer susceptibility locus. Cancer Res. 2012;72(7):1795-803.
IHCC-Szczecin Breast Cancer Study	SZBCS	Poland	Hospital-based case control study	Mixed	Prospectively ascertained cases of invasive breast cancer patients diagnosed at the Regional Oncology Hospital (Szczecin) in the years 2002, 2003, 2006 and 2007 or the University Hospital from 2002 to 2007 in Szczecin, West-Pomerania, Poland.	Unaffected, matched to cases for year of birth, sex and region; from families with negative cancer family history; controls were part of a population-based study of the 1.3 million inhabitants of West Pomerania performed in 2003 and 2004 designed to identify familial aggregations of cancer by our centre	>95% cases and 55% controls	No	315	294	**1)** Jakubowska A, Cybulski C, Szymańska A, Huzarski T, Byrski T, Gronwald J, Debniak T, Górski B, Kowalska E, Narod SA, Lubiński J. BARD1 and breast cancer in Poland. Breast Cancer Res Treat. 2008 Jan;107(1):119-22. **2)** Jakubowska A, Jaworska K, Cybulski C, Janicka A, Szymańska-Pasternak J, Lener M, Narod SA, Lubiński J; IHCC-Breast Cancer Study Group. Do BRCA1 modifiers also affect the risk of breast cancer in non-carriers? Eur J Cancer. 2009 Mar;45(5):837-42.**3)** Cybulski C, Kluźniak W, Huzarski T, Wokołorczyk D, Kashyap A, Jakubowska A, Szwiec M, Byrski T, Dębniak T, Górski B, Sopik V, Akbari MR, Sun P, Gronwald J, Narod SA, Lubiński J;
Triple Negative Breast Cancer Consortium	TNBCC	Various	See 6 studies below for details of individual studies in TNBCC.	See 6 studies below for details of individual studies in TNBCC.	See 6 studies below for details of individual studies in TNBCC.	See 6 studies below for details of individual studies in TNBCC.	See 6 studies below for details of individual studies in TNBCC.	See 6 studies below for details of individual studies in TNBCC.	152	499	See 6 studies below for details of individual studies in TNBCC.
1	*DEMOKRITOS*	*Greece*	*Hospital-based case-control study*	*Hospital-based*	*Triple negative breast cancer cases enrolled from 1997-2010 in hospitals serving geographical areas of Greece, including Athens metropolitan area, Thessaloniki, Ioannina, Patras, and Crete (Chania), in collaboration with the Hellenic Cooperative Oncology Group (HECOG).*	*Regional controls, identified between 2010-2011 from Athens and Thessaloniki, were population-based unaffected women of the same age range.*	*Unknown*	*No*			*Fostira F, et al. Prevalence of BRCA1 mutations among 403 women with triple-negative breast cancer: implications for genetic screening selection criteria: a Hellenic Cooperative Oncology Group Study. Breast Cancer Res Treat, 134(1): 353-62 (2012)*

2	GEPARSEPTO	Germany	Multicenter, prospective, randomized, open-label phase III study	Patient cohort	Patients with early primary breast cancer who are eligible for neoadjuvant chemotherapy. Recruitment at multicentres in Germany during 2011-2013.	No controls.	100%	No			**1)** Amant F, et al. (2013) Prognosis of Women With Primary Breast Cancer Diagnosed During Pregnancy: Results From an International Collaborative Study. J Clin Oncol 31 (20):2532-9. **2)** Gerber B, et al. (2014) Surgical Outcome after Neoadjuvant Chemotherapy and Bevacizumab: Results from the GeparQuinto Study (GBG 44). Ann Surg Oncol 21 (8):2517-2524. **3)** Jackisch C,et al. (2013) A randomized phase III trial comparing nanoparticle-based paclitaxel with solvent-based paclitaxel as part of neoadjuvant chemotherapy for patients with early breast cancer (GeparSepto): GBG 69. J Clin Oncol 31 (15). **4)** Untch M, et al. (2015) A randomized phase III trial comparing neoadjuvant chemotherapy with weekly nanoparticle-based paclitaxel with solvent-based paclitaxel followed by anthracyline/cyclophosphamide for patients with early breast cancer (GeparSepto); GBG 69. Cancer Res 75 (9).
3	KUMC	USA	Unknown	Unknown	Unknown	Unknown	Unknown	Unknown			Unknown
4	OSU	USA	Hospital-based case-control study	Hospital-based	Incident triple negative invasive breast cancer cases from a collection of incident breast cancer cases diagnosed in Columbus, Ohio (2006-2011).	Population-matched controls accrued through primary care clinics in the OSU medical center system (2006-2011).	Unknown	No			Unpublished
5	RPCI	USA	Hospital-based case-control study	Hospital-based	Triple negative invasive breast cancer cases from incident cases recruited to the RPCI Data Bank and Biorepository from 2006-2010.	Healthy controls identified from employee volunteers, and women recruited from community events from 2006-2010.	Unknown	No			Ambrosone CB, et al. Establishing a cancer center data bank and biorepository for multidisciplinary research. Cancer Epidemiol Biomarkers Prev 15(9): 1575-7 (2006)
6	UTMDACC	USA	Unknown	Unknown	Unknown	Unknown	Unknown	Unknown			Unknown
UK Breakthrough Generations Study	UKBGS	UK	Prospective cohort study: nested case-control study of women who had had breast cancer prior to entry into the cohort	Prospective cohort	All members who had breast cancer before entry into the Breakthrough Generations Study (cohort of 100,000+ women followed up for breast cancer, recruited from the UK during 2003-2011.	Women who had not had breast cancer, matched to cases on: age at entry to study (5 year group), year of entry into the study (≤2005, 2006, 2007, ≥2008), source of recruitment, blood sample availability and ethnicity.	All selected subjects were recruited from within the cohort study	No	470	413	Swerdlow, A.J. et al. The Breakthrough Generations Study: design of a long-term UK cohort study to investigate breast cancer aetiology. Br J Cancer 105, 911-917 (2011).
									40042	41915	

Supplementary Table S3. Participating Breast Cancer Association Consortium (BCAC) studies.

B. Asian studies

Study	Abbreviation	Country	Study design	Design category	Case definition	Control definition	Participation rates reported by Investigator	Selected familial cases	Controls	Cases with invasive BC	References
Asia Cancer Program	ACP	Thailand	Hospital-based case control study	Mixed	Cases recruited 1999-2000 and 2008-present at The National Cancer Institute (Central region), The Prince Songkla University Research Centre (South region), The HRH Princess Maha Chakri Sirindhorn Medical Centre (MSMC)-Srinakarinviroj University (Eastern region), Khon-Kaen University Cancer Centre (North-eastern region).<br>1. Women who underwent biopsy and have been pathologically diagnosed as having breast cancer.<br>2. Aged less than 71 years of age.	Controls recruited 1999-2000 and 2008-present at The National Cancer Institute (Central region), The Prince Songkla University Research Centre (South region), The HRH Princess Maha Chakri Sirindhorn Medical Centre (MSMC)-Srinakarinviroj University (Eastern region), Khon-Kaen University Cancer Centre (North-eastern region).<br>1. Women aged less than 71 years of age without cancer history of any kinds<br>2. Women who attend the out patient clinic under the minor injuries such as cuts, broken bones.<br>3. Women who are institutionalised at the hospital with diseases not related to cancer or metabolic syndromes such as diabetes, heart diseases or conditions related to gynaecology and are well enough to give information to researchers.	Cases and controls were invited to join the study and 100% of consented subjects were interviewed and provided information to the study. Over 95% of both case and control group provided blood samples.	No	634	415	Unpublished
Hospital-based Epidemiologic Research Program at Aichi Cancer Center	HERPACC	Japan	Hospital-based case control study	Mixed	Incident breast cancer cases who firstly visited Aichi Cancer Center between 2001 and 2013 and were diagnosed within 1 year from the first visit. No previous history of any type of cancer.	Controls were selected from pool of non-cancer patients who firstly visited Aichi Cancer Center between 2001-2011. Non-cancer status is defined as "having no positive finding on any of clinical/laboratory/graphical examination within 1 year from their first visit. No previous history of cancer is allowed.	Approximately 97%. As we do not have consent to access to the case-status of non-particpants, it is difficult to estimates participation rate by case-status.	No	1376	560	Kawase T et al. FGFR2 intronic polymorphisms interact with reproductive risk factors of breast cancer: results of a case control study in Japan. Int J Cancer 2009: 125:1946-1952.
Los Angeles County Asian-American Breast Cancer Case-Control Study	LAABC	USA	Population-based	Population-based	Incident cases recruited from 1995-2007 and identified from SEER cancer registries in Los Angeles County. Grouped by self-reported ethnicity.	Controls were recruited during 1995-2009 and selected from the same neighbourhood as where cancer cases resided at the time of diagnosis. Controls were frequency-matched to the cases on specific Asian ethnicities and 5-year age groups	Cases-61% of those contacted; Controls 64% were the first identified eligible control. Of the interviewed cases, 77% of interviewed cases and 81% interviewed controls gave blood/mouthwash samples	No	990	808	**1)** Wu, A.H. et al. Dietary patterns and breast cancer risk in Asian American women. Am J Clin Nutr 2009;89:1–10. **2)** Wu, A.W., et al. Birth weigth and other prenatal factors and risk of breast cancer in Asian Americans. Breast Cancer Res & Treatment 2011;130(3):917-25.
Malaysian Breast Cancer Genetic Study	MYBRCA	Malaysia	Hospital-based case control study	Mixed	Breast cancer cases identified at the Breast Cancer Clinic in University Malaya Medical Centre Jan 2003-July 2014 and Subang Jaya Medical Centre Sep 2012-Sept 2014; cases are a mixture of prevalent and incident cases. Includes hospital-based and familial series.	Controls are cancer-free individuals (37-74 years) selected from women attending mammographic screening at the same hospitals.	Not reported	No	608	746	Phuah et al., Triple-negative breast cancer and PTEN (phosphatase and tensin homologue) loss are predictors of BRCA1 germline mutations in women with early-onset and familial breast cancer, but not in women with isolated late-onset breast cancer. Breast Cancer Research 2012, 14:R142; Mariapun et al., Variants in 6q25.1 Are Associated with Mammographic Density in Malaysian Chinese Women. Cancer Epidemiol Biomarkers Prev. [illegible]
Shanghai Breast Cancer Genetic Study	SBCGS	China	Population-based case-control study, cohort study	Population-based	Newly-diagnosed breast cancer cases recruited from 1996 -2009. Cases were identified mostly from the Shanghai Cancer Registry. Some cases were identified from the Shanghai Women's Health Study.	Community controls randomly selected from the general population using the resident registry or from cancer-free cohort members in the Shanghai Women's Health Study. The controls were recruited from the same geographical region as cases during 1996-2009.	Around 85%	No	892	829	Zheng W, Long J, Gao YT, Li C, Zheng Y, Xiang YB, Wen W, Levy S, Deming SL, Haines JL, Gu K, Fair AM, Cai Q, Lu W, Shu XO. Genome-wide association study identifies a new breast cancer susceptibility locus at 6q25.1. Nature Genetics 41(3):324-8, 2009. PMCI.
Seoul Breast Cancer Study	SEBCS	Korea	Hospital-based case control study	Hospital-based	Consecutive, incident, cases from 2 hospitals in Seoul recruited 2001-2005	Healthy community controls from same catchment area and participating in annual health check-up, 2001-2005.	~ 85% of cases, 75% of controls were interviewed and provided a blood sample.	No	1129	1069	**1)** Lee, K.M. et al. Genetic polymorphisms of ataxia telangiectasia mutated an[illegible] breast cancer risk. Cancer Epidemiol. Biomarkers Prev., 14, 821-5 (2005).**2)** Han,S. et al. CASP8 polymorphisms, estrogen and progesterone receptor status, and breast cancer risk. Breast Cancer Res Treat. 110, 387-393 (2008).
Singapore Breast Cancer Cohort	SGBCC	Singapore	Hospital-based breast cancer cohort and population-based controls	Hospital-based	Living breast cancer patients diagnosed with primary insitu or invasive breast cancer at National University Hospital between 2006-2010. Cases are a mixture of prevalent and incident cases.	All community-dwelling individuals who are Singaporeans or Singaporean Permanent Residents, 21 years and older. Participants were recruited between 2006 and 2010 through word-of-mouth and personal recommendations. In some cases, recruiters also sought participants through "cold-calling" or through door-to-door invitations.<br>Exclusion criteria were a medical history of cancer, acute myocardial infarction or stroke, or major psychiatric morbidity including schizophrenia, psychotic depression, and advanced Alzheinmer's Disease.	84% of invited cases joined study. Not available for controls	No	496	384	Unpublished

IARC-Thai Breast Cancer Study	TBCS	Thailand	Hospital-based case control study	Hospital-based	Incident cases diagnosed at the National Cancer Institute (NCI) in Bangkok and Khon Kaen Hospital during the period May 2002-March 2004.	Controls were randomly selected healthy females visiting hospital patients with diseases other than breast or ovarian cancer at NCI Bangkok and Khon Kaen Hospital during the period May 2002-March 2004.	94% of cases and 73% of controls completed a questionnaire and provided a blood sample	No	253	138	Sangrajrang, S et al. Polymorphisms in three base excision repair genes and breast cancer risk in Thai women. Breast Cancer Res Treat (2008) 111:279-288
Taiwanese Breast Cancer Study	TWBCS	Taiwan	Hospital-based case control study	Hospital-based	Incident cases diagnosed & treated at 2 major teaching hospitals in Taiwan. [between March 2002 and August 2005]	Controls cancer-free individuals, randomly selected from women attending health exam. at same hospital during study period. Underwent 1-day health examination - any showing evidence cancer excluded.	>90% cases & ~ 40% of controls	No	236	776	**1)** Hsu, HM et al. Breast cancer risk is associated with genes encoding the DNA double-strand break repair Mre11/Rad50/Nbs1 complex. Cancer Epidemiol. Biomarkers Prev. 16, 2024-32 (2007). **2)** Ding, SI, et al. Genetic variants of BLM interact with RAD51 to increase breast cancer susceptibility. Carcinogenesis. 30, 43-9 (2009).
									6614	5725	

Supplementary Table S4. PCR primers for amplification of the 3’UTR of *NBS1* and the *LRRC4* gene.

Gene	Forward primer	Reverse primer
NBS1	5’-TTGTCCTAGCTACTTGGTTC-’3	5’-ATGGTCTTCAGTTTCTAGTAC-’3
LRRC4	5’-TGGGAGAGTCGTAGCTTTC-’3	5’-CGATGGGGTTGTTGCGAAG-’3

Supplementary Table S5. Clinical and demographic characteristics of breast cancer cases in the European BCAC studies.

Subgroup	N cases	rs2735383 GG+GC		rs2735383 CC		*P*-value
Age						0.64
≤ 50 years	13,362	11,866	(88.80%)	1,496	(11.20%)	
> 50 years	28,553	25,312	(88.65%)	3,241	(11.35%)	
Age at menarche						1
≤ 13 years	13,843	12,267	(88.62%)	1,576	(11.38%)	
> 13 years	8,095	7,173	(88.61%)	922	(11.39%)	
Age at menopause						0.70
≤ 50 years	7,288	6,464	(88.69%)	824	(11.31%)	
> 50 years	4,262	3,790	(88.93%)	472	(11.07%)	
Menopausal status						1
Premenopausal	7,412	6,570	(88.64%)	842	(11.36%)	
Postmenopausal	17,353	15,392	(88.70%)	1,961	(11.30%)	
Number of full-term pregnancies						0.59
≤ 2	19,722	17,518	(88.82%)	2,204	(11.18%)	
> 2	7,327	6,491	(88.59%)	836	(11.41%)	
Breast feeding						1
No	6,805	6,041	(88.77%)	764	(11.23%)	
Yes	12,709	11,278	(88.74%)	1,431	(11.26%)	
Family history						0.42
No	19,405	17,200	(88.64%)	2,205	(11.36%)	
1st degree relative with BC	4,119	3,669	(89.08%)	450	(10.92%)	
ER status						0.32
ER positive	25,959	23,003	(88.61%)	2,956	(11.39%)	
ER negative	6,774	6,032	(89.05%)	742	(10.95%)	
TN status						0.14
Non-TN	15,192	13,478	(88.72%)	1,714	(11.28%)	
TN	2,712	2,432	(89.68%)	280	(10.32%)	

N, number; BC, breast cancer; ER, estrogen receptor; TN, triple negative.
